# The Prognosis in Palliative care Study II (PiPS2): A prospective observational validation study of a prognostic tool with an embedded qualitative evaluation

**DOI:** 10.1371/journal.pone.0249297

**Published:** 2021-04-28

**Authors:** P. C. Stone, A. Kalpakidou, C. Todd, J. Griffiths, V. Keeley, K. Spencer, P. Buckle, D. Finlay, V. Vickerstaff, R. Z. Omar, on behalf of the PiPS2 investigators’ group

**Affiliations:** 1 Marie Curie Palliative Care Research Department, Division of Psychiatry, University College London (UCL), London, United Kingdom; 2 School of Health Sciences, Faculty of Biology, Medicine and Health, The University of Manchester, Manchester, United Kingdom; 3 Manchester Academic Health Science Centre, Manchester, United Kingdom; 4 Manchester University NHS Foundation Trust, Manchester, United Kingdom; 5 Palliative Medicine Department, University Hospitals of Derby and Burton NHS Foundation Trust, Derby, United Kingdom; 6 Department of Statistical Science, University College London (UCL), London, United Kingdom; University of Technology Sydney, AUSTRALIA

## Abstract

**Background:**

Prognosis in Palliative care Study (PiPS) models predict survival probabilities in advanced cancer. PiPS-A (clinical observations only) and PiPS-B (additionally requiring blood results) consist of 14- and 56-day models (PiPS-A14; PiPS-A56; PiPS-B14; PiPS-B56) to create survival risk categories: days, weeks, months. The primary aim was to compare PIPS-B risk categories against agreed multi-professional estimates of survival (AMPES) and to validate PiPS-A and PiPS-B. Secondary aims were to assess acceptability of PiPS to patients, caregivers and health professionals (HPs).

**Methods and findings:**

A national, multi-centre, prospective, observational, cohort study with nested qualitative sub-study using interviews with patients, caregivers and HPs. Validation study participants were adults with incurable cancer; with or without capacity; recently referred to community, hospital and hospice palliative care services across England and Wales. Sub-study participants were patients, caregivers and HPs. 1833 participants were recruited. PiPS-B risk categories were as accurate as AMPES [PiPS-B accuracy (910/1484; 61%); AMPES (914/1484; 61%); p = 0.851]. PiPS-B14 discrimination (C-statistic 0.837) and PiPS-B56 (0.810) were excellent. PiPS-B14 predictions were too high in the 57–74% risk group (Calibration-in-the-large [CiL] -0.202; Calibration slope [CS] 0.840). PiPS-B56 was well-calibrated (CiL 0.152; CS 0.914). PiPS-A risk categories were less accurate than AMPES (p<0.001). PiPS-A14 (C-statistic 0.825; CiL -0.037; CS 0.981) and PiPS-A56 (C-statistic 0.776; CiL 0.109; CS 0.946) had excellent or reasonably good discrimination and calibration. Interviewed patients (n = 29) and caregivers (n = 20) wanted prognostic information and considered that PiPS may aid communication. HPs (n = 32) found PiPS user-friendly and considered risk categories potentially helpful for decision-making. The need for a blood test for PiPS-B was considered a limitation.

**Conclusions:**

PiPS-B risk categories are as accurate as AMPES made by experienced doctors and nurses. PiPS-A categories are less accurate. Patients, carers and HPs regard PiPS as potentially helpful in clinical practice.

**Study registration:**

ISRCTN13688211.

## Introduction

Patients with advanced incurable cancer, their relatives and clinical teams often want to know how long patients will survive. Prognostic information can allow patients and families adequate time to prepare for the end of life [1] and can help with access to services, claiming benefits and identifying patients for inclusion in clinical trials [2]. Unlike prognoses made at diagnosis, or prior to starting systemic anti-cancer therapies (SACT) [3], those made in a palliative care context usually rely on subjective judgments of clinicians, which show a wide variation in reported accuracy [4]. The Palliative Prognostic (PaP) score, widely used in palliative cancer care, classifies patients into risk groups based on 30-day survival probabilities [5]. One limitation of PaP is that scores are heavily influenced by the weighting given to clinical predictions of survival (CPS). This can make PaP challenging to use when clinicians are unsure about survival times. The Prognosis in Palliative care Study (PiPS) predictor models were developed by members of our own group to provide prognostic estimates that do not rely on clinicians’ intuition [6]. PiPS-A14 and PiPS-A56 predict 14-day and 56-day survival in patients when no blood results are available and PiPS-B14 and PiPS-B56 predict 14-day and 56-day survival in patients when blood results are available. The outputs from each PiPS-A and PiPS-B model can be combined to produce risk categories to predict death within “days” (fewer than 14 days); “weeks” (14 to 56 days); or “months+” (greater than 56 days). The regression equations for each model and a description of the decision rules for creating risk categories are provided in on-line (S1 File). An on-line calculator is available (www.ucl.ac.uk/psychiatry/pips).

In the original development study, PiPS-A and PiPS-B models showed good discrimination. PiPS-B risk categories were more accurate than doctors’ or nurses’ survival estimates, but were not statistically significantly better than agreed multi-professional estimates of survival (AMPES) [6]. The primary objectives of the new study (PiPS2), were: to externally validate the original PiPS models [6], in a different cohort of patients, including comparison of PiPS-B risk categories against AMPES. Secondary objectives of PiPS2 were to: explore clinicians’ views about usefulness; identify barriers and facilitators to clinical use; and understand how clinicians discuss prognostic information with patients and relatives or caregivers. Further secondary objectives included evaluation of other prognostic tools, the results of which will be published separately. Only data relating to validation of PiPS-A and PiPS-B are presented here.

## Methods

The PiPS2 study was a multi-centre, prospective, validation study of the previously published PiPS prognostic models [6] in a new cohort of patients with a nested qualitative sub-study using face-to-face interviews with patients, caregivers and health professionals (HPs). The protocol has been published (ISRCTN 13688211) [7] and was approved by Yorkshire and Humber-Leeds East Research Ethics Committee (16/YH/0132).

### Sample

#### Validation study

PiPS2 involved patients from 27 UK palliative care services (S1 Table). Patients were recruited from community and hospital palliative care teams, and inpatient palliative care units. Unlike the original development study [6], the sample for PiPS2 included participants who were receiving palliative, non-hormonal SACT.

Patients who lacked capacity were included so that the sample resembled patients in clinical practice, many of whom are confused, semi-conscious, or comatose, which are all poor prognostic features. Capacity to participate was assessed by the Principal Investigator (or delegate) at each site [8]. Eligible patients with capacity were approached by a member of the clinical team, handed a Patient Information Sheet, and invited to provide written informed consent to participate. For patients without capacity a personal consultee was sought for advice. For patients with no personal consultee, the advice of a nominated consultee was sought.

*Inclusion criteria*.

1. Incurable cancer
2. 18+ years
3. Recent referral to palliative care
4. For patients with capacity, ability to read and understand Patient Information Sheet

*Exclusion criterion*. Treatment with curative intent, as determined by attending clinician.

#### Embedded qualitative study

The patient and caregiver sample comprised patients with capacity and caregivers of patients, who had been invited to participate in the PiPS2 validation study. We purposively sampled patients and caregivers so that our sample was as varied as possible and represented a wide range of views and experiences. The clinician sample comprised HPs who routinely made prognostic predictions.

### Data collection

#### Validation study

Predictor data were obtained from review of medical notes, discussion with HPs and/or directly from patients. Data required for calculation of PiPS scores are shown in Table 1.

**Table 1:** Variables required for the calculation of each prognostic score.

	PiPS-A14	PiPS-A56	PiPS-B14	PiPS-B56
**General condition**				
Pulse rate	X	X	X	X
General Health Status	X	X	X	X
Eastern Co-operative Oncology Group	X	X	X	
Abbreviated Mental Test Score	X	X	X	
**Diagnosis**				
Prostate cancer		X		X
Breast cancer		X		
Any distant metastases	X	X	X	
Bone metastases	X		X	
Liver metastases	X	X		
**Symptoms**				
Anorexia	X	X	X	
Dysphagia	X			
Dyspnoea at rest	X			
Fatigue				X
Weight loss in last month		X		
**Blood results**				
Alanine transaminase			X	
White blood count			X	X
C-reactive Protein			X	X
Platelet count			X	X
Urea			X	X
Lymphocyte count				X
Neutrophil count				X
Albumin				X
Alkaline phosphatase				X

Data were collected on the site of primary tumor and metastases, and the nature of on-going cancer treatment. Pulse rate and presence or absence of those symptoms required for calculation of PiPS scores were recorded: anorexia, dysphagia, dyspnoea, fatigue and weight loss. Abbreviated Mental Test Score (AMTS) was used to assess cognitive function [9]. To calculate PiPS scores in patients with capacity, it was only necessary to continue with AMTS until four items had been answered correctly. Patients who lacked capacity were not required to complete AMTS and were attributed scores of zero. Performance status was assessed using the Eastern Co-operative Oncology Group (ECOG) scale [10]. Global health status was rated using a 7-point clinician-rated scale with scores ranging from very poor (= 1) to excellent (= 7). For patients with capacity, blood specimens were obtained. For patients without capacity, if relevant results were available within ±72 hours of study enrolment, then they were included in analyses.

The attending doctor and nurse independently estimated survival. When they agreed, this was deemed as the AMPES. When estimates were initially discordant, the doctor and nurse discussed, and the consensus prediction was regarded as the AMPES. Clinicians were asked: to provide estimates of survival in terms of “days” (0–13 days); “weeks” (14–55 days); or “months+” (56+ days). Clinicians were also asked to provide seniority and experience.

Dates of death were obtained from NHS Digital (https://digital.nhs.uk/). If data were missing, sites were contacted to confirm survival status. Data were obtained at least five months after the last participant was recruited.

#### Embedded qualitative study

Qualitative interviews explored PiPS acceptability with patients, caregivers and HPs. Interviews used topic guides (S2 File) based on literature reviews, previous consultations with service users and recommendations for end-of-life research [11]. Topic guides were iterative to allow new themes to be explored with future participants. Interviews were conducted by the Manchester based researcher (KS) who had experience in communicating with palliative patients/discussing sensitive topics. Interviews were kept brief (< one hour), took place at a venue of participant’s choice and were audio-recorded for transcription.

### Outcomes

#### Validation study

Primary outcomes were survival (from date of enrolment), predictions of survival made by clinicians, PiPS-A and PiPS-B risk categories.

### Analysis and sample size calculation

#### Validation study

*Sample size*. To detect 5% difference (McNemar’s test) in correct predictions between PiPS-B risk categories and AMPES [6], 1267 patients with complete PiPS-B data were required (80% power; 5% significance). Assuming 25% of participants would lack capacity (thereby unable to provide PiPS-B data), and assuming 5% missing data, we estimated a sample of 1778 would be required.

It has been recommended that validation data for risk models should have at least 100 events [12]. There is no guidance on sample size calculation for multi-centre prognostic validation studies where there is potential of clustering. To be conservative, we inflated number of events to validate prognostic models to 150. Assuming an event rate of 17.8%, based on the original study, we estimated 843 patients would be required to validate PiPS-B risk categories. Therefore, the proposed sample size for the primary outcome was considered to be adequate to also validate PiPS-A and PiPS-B.

*Statistical analyses*. Model discrimination was assessed using the C-statistic which measures a risk model’s ability to discriminate between those who experience the outcome of interest (survive a given number of days) and those who die. The C-statistic is calculated by considering all possible pairs of patients in the study and estimating the proportion of pairs in which the probability predicted by the model for survival is higher for the patient who actually survived compared to the patient who died. A value of 1 indicates the model has perfect discrimination, while a value of 0.5 indicates the model discriminates no better than chance [13].

Model calibration was assessed using calibration slope (CS) and calibration in the large (CiL) [14] based on a logistic model. The calibration slope is a measure of agreement between the observed and predicted risk of the outcome across the whole range of predicted values obtained from the model and values close to 1 indicate good calibration. A slope <1 indicates that some predictions are too extreme (that low risks are underestimated, and high risks are overestimated) and a slope >1 indicates the range of predicted probabilities is too narrow. Calibration-in-the-large measures the extent that predictions are systematically too low or too high. It compares the mean of all predicted risks with the mean observed risk and should ideally be 0 [13].

Calibration of PiPS-B14 and PiPS-B56 was also assessed by comparing observed and predicted proportions of events graphically for each decile of predicted risk. Overall proportion of deaths (calculated combining days, weeks and months+ risk category predictions) predicted correctly by PiPS-B risk categories was compared with overall proportion of deaths predicted correctly by clinicians using McNemar’s test. For secondary analyses, significance level for McNemar’s tests was amended (0·05/3 = 0·0167) using a Bonferroni adjustment to account for multiple comparisons. Bias due to missing data was investigated and multiple imputation using chained equations was used to impute missing predictor values. Statistical analyses were performed using Stata v14 [15]. The original PiPS study did not include patients receiving disease-modifying treatments expected to prolong survival, whereas not all such patients were excluded from PiPS2. We therefore chose to validate PiPS both in all eligible participants and in the sub-group who were no longer receiving non-hormonal SACT.

#### Embedded qualitative study

Sample size was determined by data saturation. Interview transcripts were analysed using the five stages of Framework Analysis facilitated by NVivo 10 (https://www.qsrinternational.com/nvivo) [16]. First, the research team became immersed in the data. Second, a thematic framework was developed based on the topic guide. Thirdly, transcripts were indexed (coded) line-by-line using the thematic framework, but remaining open to emerging themes. Fourthly, data were entered into a chart so that coded extracts could be attributed to individual participants. Finally, participants’ views were compared and contrasted, and data were presented schematically (mapping). Rival explanations were explored. An iterative and inductive approach to analysis was followed with data analysis occurring alongside data collection. The qualitative research team met regularly to discuss the development of codes, themes, categories and theories about phenomena being studied.

## Results

### Validation study

A total of 17014 patients were screened at 27 sites (August 2016-April 2018); 3299 were eligible and invited to participate; 1833 (1610 with; 223 without capacity) were enrolled. There were no significant differences in age or gender between patients who agreed or did not agree to participate. Patients who declined consent were not obliged to provide reasons. The most common explanations volunteered were: fatigue; distress; malaise; or competing priorities. Median survival of participants from enrolment was 45 days (IQ Range 16 to 140). Proportion of participants not receiving non-hormonal SACT was 1603/1833 (87%). There were complete data on 89% (1484/1671) of participants, who were potentially available to have PiPS-B risk categories calculated (i.e. those with capacity and those without capacity with a recent blood test). Only minor differences were found between results obtained from analyzing complete and imputed data (S3 File), and so only complete data results are presented here.

Participant characteristics are shown in Table 2.

**Table 2:** Participant characteristics.

Variable	
Age (years); mean (SD); n = 1832*	70·2 (11·9)
Gender; n (%); n = 1832*	
Male	938 (51·2)
Female	894 (48·8)
**Location; n (%)**	
Inpatient Palliative Care Unit	1241 (67·7)
Community Palliative Care Team	468 (25·5)
Hospital Palliative Care Team	124 (6·8)
Site of Primary tumor**; n (%)	
Lung	362 (19·8)
Upper GI tract	337 (18·4)
Head and neck	280 (15·3)
Prostate	160 (8·7)
Breast	146 (8·0)
Gynaecological	133 (7·3)
Other	123 (6·7)
Urological (bladder, testes, renal)	112 (6·1)
Lower GI tract	81 (4·4)
Haematological	70 (3·8)
Unknown	45 (2·5)
Neurological	38 (2·1)
Rare tumor	27 (1·5)
**Site of metastatic diseases; n (%)**	
Bone	555 (30·3)
Liver	538 (29·4)
Nodal	516 (28·2)
Lung	477 (26·0)
Other	353 (19·3)
None	279 (15·2)
Brain	134 (7·3)
Pleural effusion	98 (5·4)
Ascites	95 (5·2)
Adrenal	79 (4·3)
Unknown	60 (3·3)
Skin	36 (2·0)
Renal	20 (1·1)
**Currently receiving tumor therapy; yes n (%)**	391 (21·3)
**If yes, type of therapy**:	
Chemotherapy	190 (48·6)
Radiotherapy	118 (30·2)
Hormone therapy	76 (19·4)
Other tumor directed therapy (e.g. immunotherapy)	42 (10·7)
**Capacity to consent; n (%)**	1610 (87·8)
**Abbreviated Mental Test Score (AMTS); n = 1826**	
Less than 4	208 (11·4)
Greater or equal 4	1618 (88·6)
**Presence or absence of symptoms included in prognostic scores**	
Anorexia; yes; n = 1830	968 (52·9)
Dysphagia; yes; n = 1830	554 (30·3)
Dyspnoea; yes; n = 1831	652 (35·6)
Fatigue; yes; n = 1831	1617 (88·3)
Lost weight; yes; n = 1831	1194 (65·2)
**Clinical assessments**	
Pulse rate; beats/min; mean (SD); n = 1817	82·2 (14·7)
**Eastern Co-operative Oncology Group score (ECOG) Performance status; n = 1831**	
Grade 0	15 (0·8)
Grade 1	202 (11·0)
Grade 2	520 (28·4)
Grade 3	822 (44·9)
Grade 4	272 (14·9)
**Global health status (overall health); n (%); n = 1823**	
1 (Very poor)	144 (7·9)
2	414 (22·7)
3	680 (37·3)
4	348 (19·1)
5	180 (9·9)
6	49 (2·7)
7 (Excellent)	8 (0·4)
**Full blood count**	**Mean (SD)**
White blood count (x10^9^/L); n = 1602	11·3 (11·2)
Lymphocyte count (x10^9^/L); n = 1596	1·2 (2·0)
Neutrophil count (x10^9^/L); n = 1600	8·8 (6·2)
Platelets (x10^9^/L); n = 1601	312·9 (147·6)
**Biochemistry**	
Urea (mmol/L); n = 1601	8·0 (6·4)
Albumin (g/L); n = 1600	30·1 (7·0)
Alkaline phosphatase (U/L); n = 1587	231·7 (319·9)
Alanine transaminase (U/L)); n = 1581	33·3 (71·7)
C reactive protein (mg/L)); n = 1565	68·6 (73·5)

* One participant preferred not to say.

** 73 participants had more than one primary tumor.

#### PiPS

Discrimination and calibration of PiPS-A and PiPS-B, 14-day and 56-day models including the sub-group of participants no longer receiving non-hormonal SACT are shown in Table 3. All of the PiPS models showed good or excellent discrimination (C-Index ranging from 0.772 to 0.837).

**Table 3:** Discrimination and calibration of PiPS-A and PiPS-B 14-day and 56-day models in patients receiving or not receiving non-hormonal SACT.

	n	C-statistic/index^†^ (95% CI)	Calibration in the large (95% CI)*	Calibration slope (95% CI) *
**PiPS-A model (all patients)**				
PiPS-A14	1802	0·825 (0·803 to 0·848)	-0·037 (-0·168 to 0·095)	0·981 (0·872 to 1·09)
PiPS-A56	1803	0·776 (0·755 to 0·797)	0·109 (0·002 to 0·215)	0·946 (0·842 to 1·05)
**PiPS-A model (not on non-hormonal SACT)**				
PiPS-A14	1573	0.820 (0.795 to 0.844)	-0.077 (-0.215 to 0.061)	0.967 (0.853 to 1.081)
PiPS-A56	1574	0.772 (0.749 to 0.795)	-0.035 (-0.150 to 0.080)	0.932 (0.821 to 1.044)
**PiPS-B model (all patients)**				
PiPS-B14	1498	0·837 (0·810 to 0·863)	-0·202 (-0·364 to -0·039)	0·840 (0·730 to 0·950)
PiPS-B56	1498	0·810 (0·788 to 0·832)	0·152 (0·030 to 0·273)	0·914 (0·808 to 1·02)
**PiPS-B model (not on non-hormonal SACT)**				
PiPS-B14	1300	0.832 (0.803 to 0.860)	-0.218 (-0.389 to -0.047)	0.853 (0.735 to 0.971)
PiPS-B56	1299	0.805 (0.781 to 0.829)	0.031 (-0.099 to 0.161)	0.901 (0.788 to 1.015)

* To calculate the calibration estimates for the PiPS-B14 model one participant with an outlying value for their estimated prognostic index was removed.

^†^ The C-statistic gives the probability that a randomly selected patient who survived had a higher prediction than a patient who had died.

**Fig 1:**
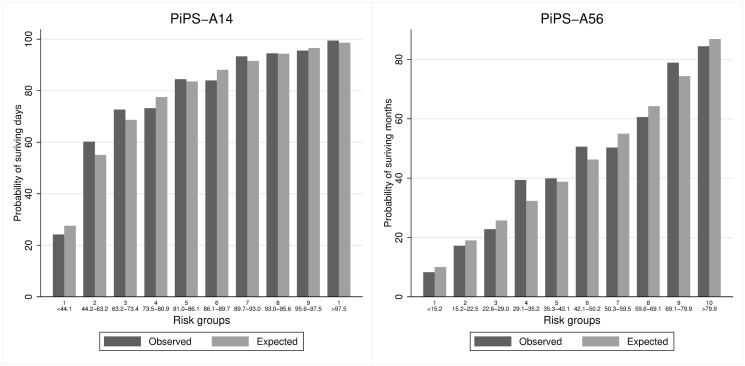
PiPS-A all patients. Observed and predicted proportion of events using PiPS-A14 and PiPS-A56 in all patients. Vertical bars represent observed (dark grey) and model-based predicted (light grey) probabilities of surviving either days (left) or months (right). The risk groups were created using the model-based predicted probabilities with an equal number of participants being allocated into each risk group. The predicted probabilities used for each risk group are shown. These groups are selected for the purpose of validation rather than clinical decision making. PiPS-A14: n = 1802; Proportion of events = 1407/1802 (78.1%). PiPS-A56: n = 1803; Proportion of events = 815/1803 (45.2%).

**Fig 2:**
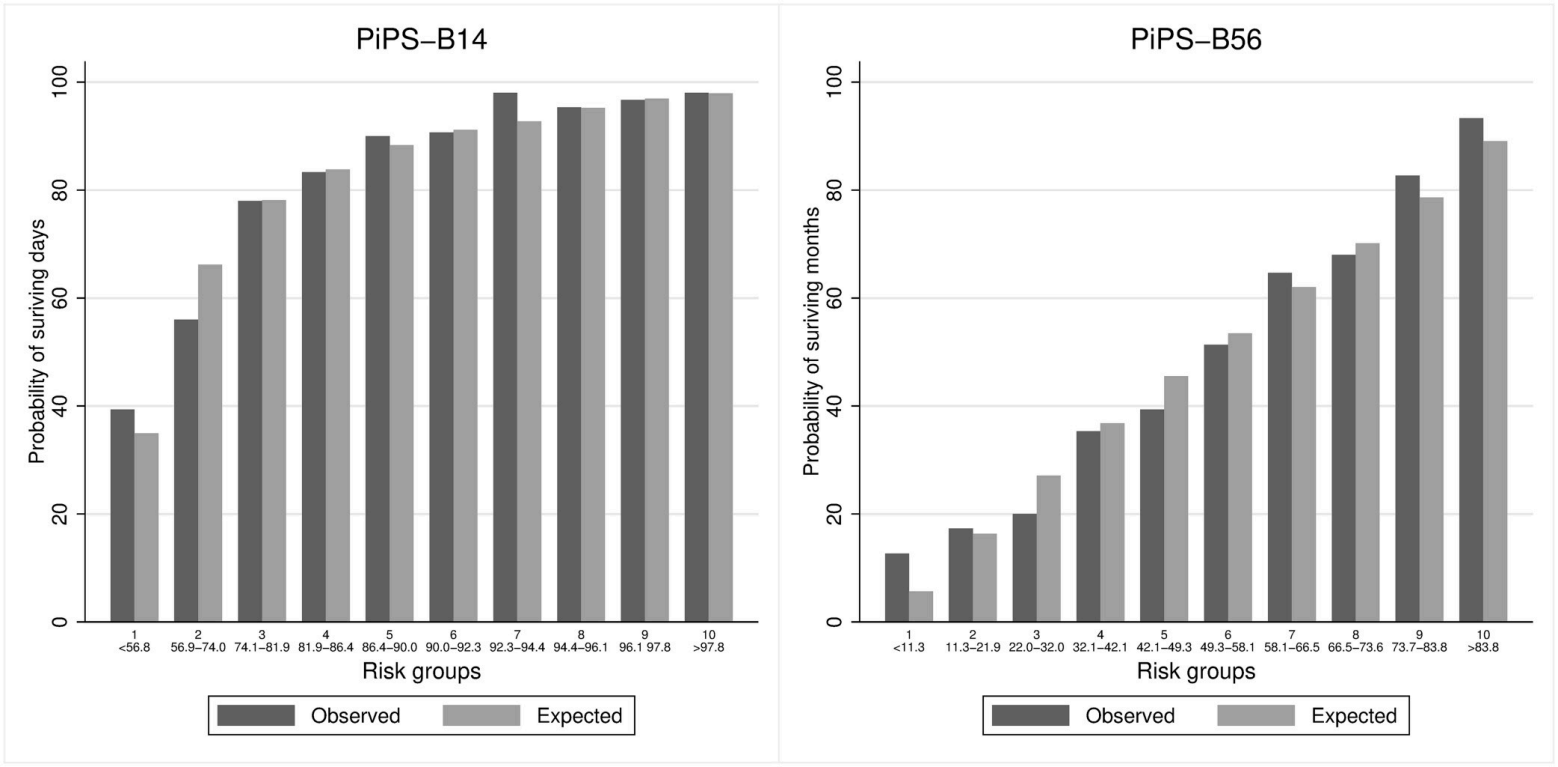
PiPS-B all patients. Observed and predicted proportion of events using PiPS-B14 and PiPS-B56 in all patients. Vertical bars represent observed (dark grey) and model-based predicted (light grey) probabilities of surviving either days (left) or months (right). The risk groups were created using the model-based predicted probabilities with an equal number of participants being allocated into each risk group. The predicted probabilities used for each risk group are shown. These groups are selected for the purpose of validation rather than clinical decision making. PiPS-B14: n = 1497; Proportion of events = 1238/1497 (82·7%). One participant was removed from this analysis as their PiPS-B14 value was an outlier. PiPS-B56: n = 1498; Proportion of events = 727/1498 (48·5%).

**Fig 3:**
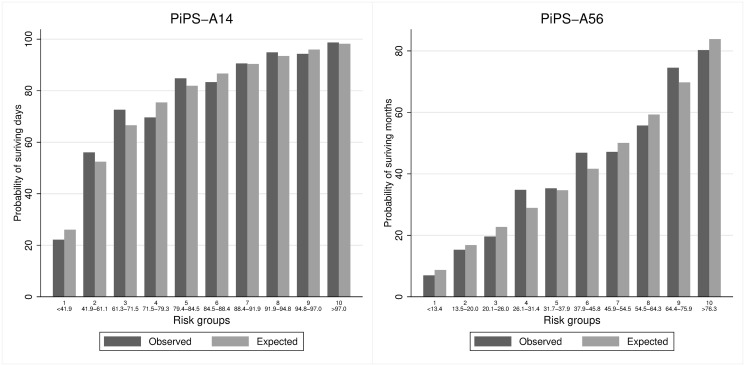
PiPS-A patients receiving non-hormonal SACT. Observed and predicted proportion of events using PiPS-A14 and PiPS-A56 in patients receiving non-hormonal SACT. Vertical bars represent observed (dark grey) and model-based predicted (light grey) probabilities of surviving either days (left) or months (right). The risk groups were created using the model-based predicted probabilities with an equal number of participants being allocated into each risk group. The predicted probabilities used for each risk group are shown. These groups are selected for the purpose of validation rather than clinical decision making. PiPS-A14: n = 1573; Proportion of events = 1206/1573 (76.7%). PiPS-A56: n = 1574; Proportion of events = 655/1574 (41.6%).

**Fig 4:**
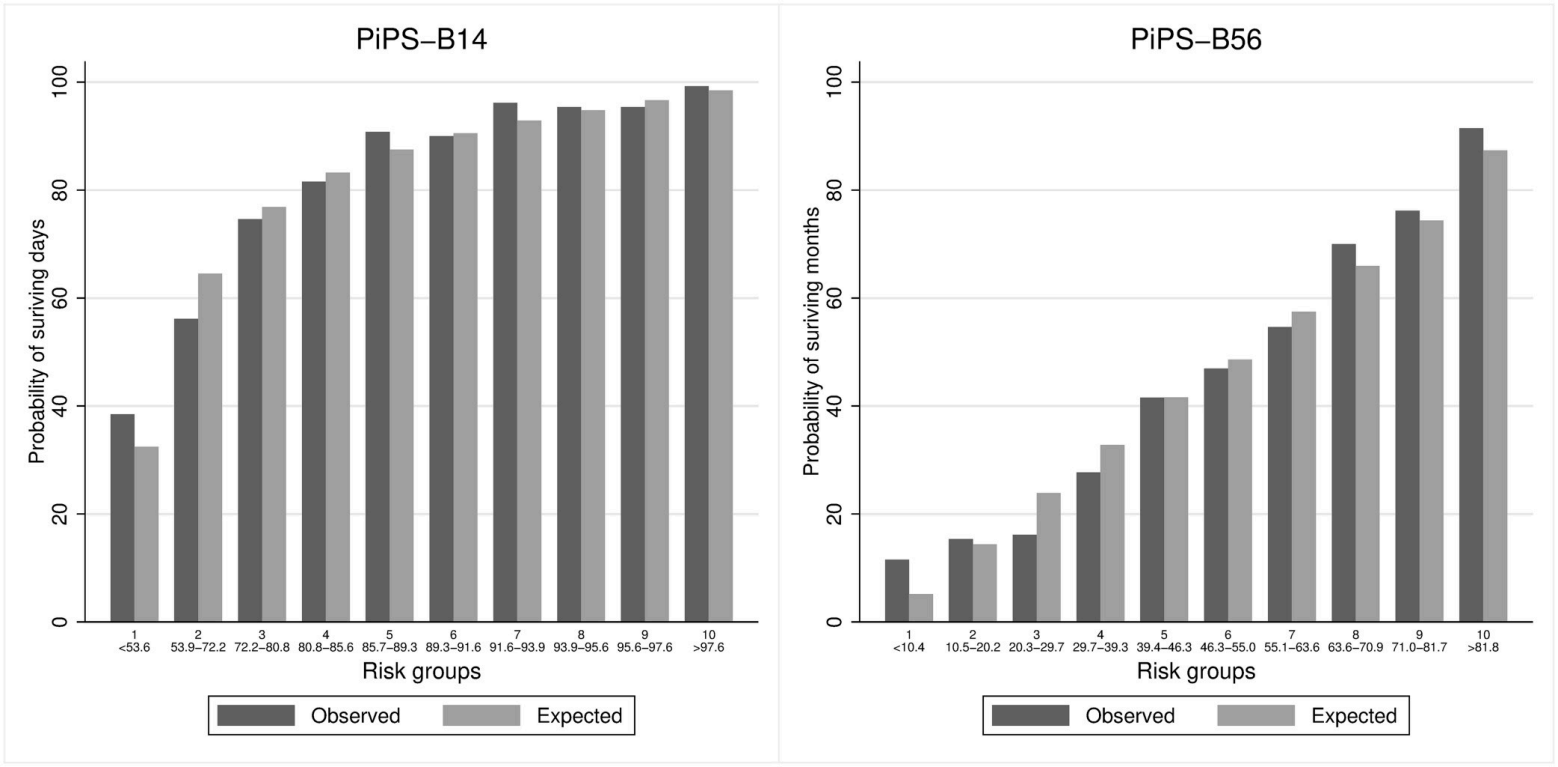
PiPS-B patients receiving non-hormonal SACT. Observed and predicted proportion of events using PiPS-B14 and PiPS-B56 in patients receiving non-hormonal SACT. Vertical bars represent observed (dark grey) and model-based predicted (light grey) probabilities of surviving either days (left) or months (right). The risk groups were created using the model-based predicted probabilities with an equal number of participants being allocated into each risk group. The predicted probabilities used for each risk group are shown. These groups are selected for the purpose of validation rather than clinical decision making. PiPS-B14: n = 1300; Proportion of events = 1063/1300 (81.8%). PiPS-B56: n = 1299; Proportion of events = 586/1299 (45.1).

Figs 1–4 suggest that PiPS-A14, PiPS-A56 and PiPS-B56 models were well-calibrated. PiPS-B14 showed some degree of over fitting, with predictions slightly higher for 57%-74% risk group (CiL -0.202: -0.364 to -0.039; CS 0.840: 0.730 to 0.950).

PiPS-A and PiPS-B 14-day and 56-day model predictions were combined to create risk categories representing whether patients would survive for “days”, “weeks” or “months” (S2). The accuracy of predictions based on PiPS-A and PiPS-B risk categories, compared against accuracy of AMPES is shown in Table 4.

**Table 4:** Performance of PiPS-A and PiPS-B risk categories compared to an agreed multi-professional estimates of survival (AMPES) in patients receiving or not receiving non-hormonal SACT.

PiPS-A risk categories in all patients ^a^			
	AMPES compared to overall observed deaths		
PiPS-A predictions compared to observed deaths	Number (%) of patients when AMPES was correct	Number (%) of patients when AMPES was incorrect	Total
Number of patients when PiPS-A prediction was correct	762 (42·3%)	250 (13·9%)	1012
Number of patients when PiPS-A prediction was incorrect	355 (19·7%)	435 (24.1%)	790
Total	1117	685	1802
PiPS-A risk categories in patients not receiving non-hormonal SACT ^b^			
	AMPES compared to overall observed deaths		
PiPS-A predictions compared to observed deaths	Number (%) of patients when AMPES was correct	Number (%) of patients when AMPES was incorrect	Total
Number of patients when PiPS-A prediction was correct	652 (41.4%)	227 (14.4%)	879
Number of patients when PiPS-A prediction was incorrect	297 (18.9%)	397 (25.2%)	694
Total	949	624	1573
PiPS-B risk categories in all patients ^c^			
	AMPES compared to overall observed deaths		
PiPS-B predictions compared to observed deaths	Number (%) of patients when AMPES was correct	Number (%) of patients when AMPES was incorrect	
Number of patients when PiPS-B prediction was correct	685 (46·2%)	225 (15·2%)	910
Number of patients when PiPS-B prediction was incorrect	229 (15·4%)	345 (23·2%)	574
Total	914	570	1484
PiPS-B risk categories in patients not receiving non-hormonal SACT ^d^			
	AMPES compared to overall observed deaths		
PiPS-B predictions compared to observed deaths	Number (%) of patients when AMPES was correct	Number (%) of patients when AMPES was incorrect	
Number of patients when PiPS-B prediction was correct	577 (44.8%)	205 (15.9%)	782
Number of patients when PiPS-B prediction was incorrect	194 (15.1%)	311 (24.2%)	505
Total	771	516	1287

^a^ Percentage of correct AMPES significantly (p<0.001) better than percentage of correct PiPS-A risk category predictions.

^b^ Percentage of correct AMPES significantly (p = 0.002) better than percentage of correct PiPS-A risk category predictions.

^c^ Percentage of correct AMPES not significantly (p = 0.851) different than percentage of correct PiPS-B risk category predictions.

^d^ Percentage of correct AMPES not significantly (p = 0.582) different than percentage of correct PiPS-B risk category predictions.

The majority of AMPES were made by palliative care doctors (360/431 = 85.5%) and nurses (755/771 = 98.3%) with a median (IQ range) of 9 (5–20) and 19 (9–30) years’ of professional experience respectively. There were no statistically significant differences between percentage of correct AMPES and percentage of correct predictions based on PiPS-B risk categories when compared to all observed deaths, in either the whole sample or in the sub-group no longer receiving non-hormonal SACT. In contrast, a statistically significantly higher percentage of AMPES were correct compared to PiPS-A risk categories, in both samples.

### Qualitative study

Interviews were held with 29 patients, 20 caregivers and 32 clinicians. The majority of patients (25/29; 86%) and caregivers (17/20; 85%) were recruited from two hospices in one city. Details about the analysis are available as S4 File. Illustrative quotes are shown in Table 5.

**Table 5:** Illustrative quotes from the qualitative study.

**Patients and Caregivers’ quotes**
***Desire for detailed patient prognostic information***
“Whenever I have asked doctors about my prognosis nearly everybody’s been vague, to be honest. I know that I’m going, but I want to know, even now, have I got a couple of weeks, a couple of months, the end of the year? I certainly know it isn’t anything further than that, but nobody dare tell me. I think doctors tend to be optimistic, I’d rather they would be more realistic.” Male patient, aged 63 (hospice), (ID P8).
“Nobody told him whether he’d got a week, a month, or a year to live. They [the oncologist] just said the cancer was bad and we were just in shock because nobody could tell us what time my husband had left, the oncologists are reluctant to say, but I think as individuals you need some idea.” Female caregiver, aged 56, (community), (ID C18).
“I would rather not know, when I am going to go… I’ve lived a long life and I think I’d probably be more anxious, if I knew exactly when.” Female patient, aged 92 (hospice), (ID P21).
***Acceptability of PiPS predictor models***
“I think developing this tool is massively needed. I’ve found the hardest part of going through all of this is the not knowing. I’m always asking, can you tell me how long, and everybody says the same thing, sadly, we can’t. We were given a rough estimation, like obviously 12 months being the longest survival rate from the oncologist, and we are actually past that now.” Female caregiver, aged 35 (hospice), (ID C7).
“I think the tool would be useful to help doctors start that sort of conversation about time left for people, I have felt that it is treated as a big secret as if they [doctors] feel embarrassed to tell you or don’t know what to say. But I think if you’ve got something saying, look, we’ve had a look at this tool and it says maybe it’s months not years, you know a bit of power to your elbow is always useful, isn’t it?” Female, aged 61, (hospice), (ID P15).
***Presentation of sensitive information***
“Rather than being given statistical information about my husband’s time left, I’d rather doctors say to me, it’s days or weeks, rather than there’s a 50 per cent chance he will still be here in 2 months. It’s just clearer to understand that way for me.” Female caregiver, aged 35, (hospice), (ID C7).
**Clinicians’ quotes**
***Challenges and difficulties with predicting length of survival***
“In the past I’ve found it extremely difficult to give prognostic information, especially when working in a hospital as it seems more unpredictable and, you are not really working in a specific palliative care environment, you do sometimes feel a bit lost with these kinds of conversations.” Trainee doctor (ID H15).
“I find predicting length of survival extremely difficult. I think once there’s a change in somebody’s health condition it’s certainly a lot easier, because then you’ve got a reason to suspect that they may well be deteriorating. If somebody continues in a stable disease phase without the presence of imaging, I think it’s difficult to prognosticate.” Consultant (palliative care), (ID H24).
***Language used when discussing prognosis***
“I tend to be quite guarded and careful about how I answer that question, and usually probably far too vague for their liking, mainly because I’ve seen the negative effects of people having been given a very clear timescale, often from a hospital clinic they’re given x number of months, and more often than not they might exceed that and then they feel like they’re living on borrowed time, and actually psychologically for them and their family that’s often more damaging.” GP, (ID H9).
“I don’t ever use numbers, because it fixes people’s focus onto a particular time scale, and rather than making what they can and enjoying each day for its own right, and, you know, having an eye to the things that they need to be sorting out. I think that’s the sense of a much more vague timeline from that point of view is far more helpful for most people.” Consultant (palliative care), (ID H31).
***Reasons for over-estimating prognosis***
“It’s about all sorts of complex reasons why doctors may over estimate prognosis really, It’s trying not to upset patients, not be a failing doctor, trying to understand what death and prognosis means to patients and what else is going on in people’s lives”. Specialty Trainee Doctor (ID H17).
“It’s not that clinicians are over estimating prognosis it may be that sometimes, we can see that a patient is operating at a high level of denial, we wouldn’t necessarily challenge if we thought that was a useful coping mechanism for them, any challenge may cause stress and harm to the patient, but it might be that we then have to subtly bring along the patient and family’s understanding over time to try and prepare them for what’s going to happen”. Consultant (palliative care), (ID H14).
***Clinicians’ acceptability of PiPS models***
“I think we’re a little bit uncomfortable when we are asked the question about what time is left, because we know that it’s an estimate? We don’t want to be completely wrong, I suppose, and yet we understand that something sudden can happen at any point, can’t it? So, I think the PiPS tool perhaps gives you more confidence in making a prediction.” Consultant oncologist, (ID H20).
We know that we can never be a hundred percent sure, but what this tool does is gives you a bit more confidence. Maybe more doctors would have the conversation if they felt more confident about what they were saying, and actually that would be much better if doctors started talking about it more, that would be a really good thing.” Consultant (palliative care), (ID H14).
***Facilitators for use in clinical practice***
“It would be interesting for junior staff like me to use PIPS that are new to palliative care. Say, okay, for your first month in your job, try and plug in the details of the patients that we’ve actually got the relevant details for, and then just get a feel yourself on how that’s matching up to your own reality and clinical judgement of what time patients have left”. Trainee doctor, F1 (ID H6).
“Even if it’s no more accurate than clinicians’ estimate, I would still use it, especially in the hospital setting. The reason being I think one of the things…or at least from a palliative care point of view, is that it could aid any MDT discussions as sometimes it is difficult to convince other clinicians about a patient’s prognosis. If we can use PiPS, then at least we could use this as an evidence base to say why we think the particular patient has got weeks or months”. Consultant (palliative care), (ID H30).
***Barriers to use in clinical practice***
“I am not sure how ethical it is to be taking bloods with patients that have advanced cancer, especially if I am seeing them at home. It is about making them comfortable not sticking them with needles.” GP, (ID H4).
“We’re saturated at the moment in primary care so to have to do something else like complete and run a PiPS estimate could be time consuming. Also then you’ve got to find time to give patients the result.” GP, (ID H11).
“All the statistics doesn’t fit well with everyone and I just would always trust my own clinical judgement because that’s what I’ve always done. And if using a tool doesn’t benefit the patient in any way then it won’t be used.” GP, (ID number, H9).

The majority of patient and caregivers clearly expressed a desire for detailed prognostic information, but often reported that clinicians were vague, over-optimistic and unwilling to deliver accurate information about length of survival. The main reason for wanting detailed information was to put finances in order and make funeral plans. All patients and caregivers considered PiPS was: acceptable for use in clinical practice; a potentially useful aid for predicting life expectancy; and helpful for initiating sensitive conversations with patients and caregivers. Participants confirmed that life expectancy expressed in terms of days, weeks or months was most meaningful.

Clinicians reported finding estimating length of survival complex and often challenging, and the process of conveying prognostic information to patients and caregivers to be difficult and uncomfortable. Clinicians explained they avoided giving specific timeframes in discussions because they did not know or did not want the discussion to have a negative impact on patient or caregiver. They admitted being vague with patients and caregivers, and considered that PiPS might be a useful communication aid for conveying prognostic information.

Clinicians considered PiPS might act as an educational training tool, especially for less experienced staff. They further commented on how PiPS might help inform decision-making, in relation to treatment options, discharge planning and admission to hospices, or when commissioning care. Clinicians said that, even if PiPS risk categories were no more accurate than their own estimates, they would still regard them as potentially beneficial tools that could help improve confidence in making survival predictions.

Clinicians identified a number of barriers to using PiPS in clinical practice. The need for a blood test was a potential barrier to using PiPS-B. Two of the doctors considered introducing PiPS into clinical practice could be time-consuming, both in completing the tool and finding time to communicate results to patients and families. Other barriers related to clinicians preferring to rely on their own clinical judgement, or wishing to avoid prognostic discussions with patients and caregivers.

## Discussion

In the PiPS2 study, the previously published PiPS-A and PiPS-B models for predicting 14-day and 56-day survival [6] showed good or excellent discrimination. The PiPS-A risk categories (“days”, “weeks” and “months+”) were significantly less accurate than AMPES, and should not be used in clinical practice in their current form except in a research setting. However, the PiPS-B risk categories were as accurate as AMPES at identifying patients who were expected to live for “days”, “weeks” or “months+”. Our qualitative work confirms that, even though PiPS-B risk categories were no more accurate than AMPES, they may still be a valuable addition to clinical practice because they could provide some objectivity and reproducibility into an area that is currently dominated by intuition.

PiPS2 is one of the largest prospective palliative care studies undertaken in the UK. The study was powered to demonstrate a difference between the accuracy of PiPS-B risk categories and AMPES. Previous prognostic studies have simply validated various prognostic tools statistically and have reported their discrimination, calibration and accuracy. However, in clinical practice “usual care” relies on clinician predictions. Therefore, it is important that newly proposed prognostic tools should be at least as accurate as this before being considered for adoption into clinical practice. Our qualitative sub-study was a great strength because it allowed a greater understanding of the perceived value of these tools to patients, their families and the health care professionals looking after them. One potential limitation of this study is that PiPS is only designed to be used in patients with advanced cancer. There is an increasing recognition of the need to widen the access to palliative care services to more patients with non-malignant disease. Nonetheless, it remains the case that cancer patients currently make up the majority of palliative care referrals and would benefit from improved prognostication. Our qualitative research was limited by the relatively few views that were represented from patients who did not want to participate, in the PiPS2 quantitative study (and so who may have had less positive opinions about PiPS) and from community patients (whose views may have differed from hospital or hospice-based participants). Another potential limitation was that the same research fellow recruited patients to both the quantitative and qualitative studies, and conducted the qualitative interviews (in the Greater Manchester area). There was therefore a risk of respondents reporting overly positive experiences. However, methodologically (and ethically) it was appropriate for the same researcher to recruit to the nested study because of the need to purposively sample according to certain characteristics. Participants were gravely ill and recruitment needed to be as sensitive as possible. Also, while KS was part of the research team for PiPS2, she was not involved in the original development of PiPS, and had no vested interest in a positive or negative response from patients.

In the last five years, two further groups have validated PiPS. Baba and colleagues [17] studied 2426 Japanese palliative cancer patients, some of whom were receiving palliative chemotherapy. They reported PiPS performed as well as in the original study [6], but they did not compare its accuracy to that of clinicians. The only previous study to have done so [18], involving 202 Korean cancer patients, reported PiPS risk categories were more accurate than doctors’ estimates of survival. However, this study was limited by being relatively small (n = 202) and because it used doctors’ uni-professional survival estimates rather than AMPES as the comparator.

There were some differences between participants in the original PiPS development study and in PiPS2. In the original study, median survival of participants was 34 days and none were receiving disease-modifying treatments, in PiPS2 it was 45 days and 12.5% were receiving non-hormonal SACT. This may explain the small degree of model over-fitting that we found and suggests that some recalibration may be required to use these models in palliative patients who are still receiving non-hormonal SACT. Baba and colleagues [17] previously reported that PiPS performed as well in patients who were or were not receiving palliative cancer treatments. In the sub-group analysis for this study we found that excluding participants receiving palliative treatments did not make any substantial differences to our results, although calibration of PiPS-A56 and PiPS-B56 both improved somewhat. The use of PiPS-B risk categories in this sample resulted in a lower proportion of incorrect prognoses than when applied to the whole sample (and fewer incorrect prognoses compared to AMPES). However, the difference in overall accuracy between PiPS-B risk categories and AMPES remained non-significant (p = 0.582).

There is evidence that AMPES are more accurate than predictions made by staff acting alone [19]. However, it is not always convenient or practical to obtain a second opinion when making a prognosis. It may also be more demanding in terms of time and resources to do so. PiPS-B may provide clinicians with added confidence in their prognostic predictions, and could act as a “second opinion” in situations when one is not readily available. In this study, AMPES were usually estimated by experienced palliative care staff who may have been more accurate than less experienced individuals. Therefore, PiPS-B could be of particular value in less specialist health care settings. PiPS-B could also provide more objective criteria by which to determine entry to clinical trials for palliative care patients. Scores may help to describe the case-mix of patients and facilitate comparison between clinical services. PiPS may also help to standardise communication between professionals and foster greater trust in the objectiveness of prognostic estimates between referrers to, and providers of, palliative care services. Certain benefits and services are influenced by clinical predictions of survival but clinician confidence in their own predictions is low and this may be a barrier to access. Routine use of validated prognostic tools like PiPS may improve access to such services.

The PiPS prognostic tools are freely available to use as an on-line calculator (www.ucl.ac.uk/psychiatry/pips). However, it is important to note that, since the tools are still being evaluated and refined, the calculator should only be used and interpreted by palliative care physicians and other suitably qualified health professionals. The calculator should not be used as a replacement for clinical judgement and nor should it be used by patients alone.

Although PiPS-B risk categories are as accurate as AMPES, further research is needed to determine whether their routine use could improve outcomes for palliative care patients. This will probably require a large multi-centre randomised controlled trial comparing usual practice (using clinician predictions) against enhanced care (additionally incorporating PiPS-B predictions). One of the difficulties with the design of such a study will be identifying and measuring those clinical outcomes which are most likely to be affected by better prognostication. Until a prognostic tool has been shown to improve clinically relevant outcomes it is unlikely to be adopted into practice, this is one of the reasons why many palliative prognostic tools exist, but few are routinely used. It is possible that in other clinical settings (e.g. primary care or acute oncology), or among other practitioners (e.g. junior doctors or nurses), the clinician predictions of survival may be less accurate. In those circumstances, PiPS-B may have a greater role as an aid to prognostication. Further research could also attempt to optimise the performance of the PiPS tools, either by adjustment of the “decision rules”, recalibration or a combination of the two.

## Supporting information

S1 File Regression equations and decision rules. (DOCX)

S2 File Topic guides. (DOCX)

S3 File Multiple imputation analysis for PiPS-B model. (DOCX)

S4 File Qualitative sub-study summary. (DOCX)

S1 Table Participating units and principal investigators. (DOCX)

S1 Appendix PiPS2 investigators’ group—Names of authors for referencing in PubMed. (DOCX)
