# Supplementary material for: The Prognosis in Palliative care Study II (PiPS2): A prospective observational validation study of a prognostic tool with an embedded qualitative evaluation
Source: PLoS One. 2021 Apr 28;16(4):e0249297. doi: 10.1371/journal.pone.0249297 (PMC8081241; doi:10.1371/journal.pone.0249297)
Supplement: S1 File — (DOCX) [file pone.0249297.s001.docx]

# S1 File. Regression equations and decision rules

For both PiPS-A and PiPS-B, two separate models have been developed to predict the two-week (14 day) and two month (56 day) survival of patients (thus generating three prognostic categories; less than two weeks, two weeks to two months and greater than two months). The weeks and months models include different sets of predictors. For both models (weeks and months), if the predicted probability of survival exceeded 50% for a patient, then the patient was classified to have survived beyond that time point (thus for example if the predicted probability from PIPS 56-day models was ≥50%, then the patient was predicted to survive months). Otherwise, it was assumed that the patient did not survive to that time point. If the predicted survival probability from the 14-day models was ≥50%, but the predicted survival probability from the 56-day models was <50%, then the PiPS outcome would be that the patient was predicted to die in “weeks”. Finally, if the predictions of the 14-day and 56-day models were discordant (e.g. 14-day model survival probability ≥50%, but 56-day model survival probability <50%) then the patient was also predicted to survive for “weeks”.

**PiPS-A14 model**

The PiPS-A14 log odds are

$${LO}_{A_{14}} = 3\cdot82 + 1\cdot273amts -0\cdot023pulse -0\cdot498distant_{mets} - 0\cdot538mets_{liver} - 0\cdot563ecog + 0\cdot449overall\_health - 0\cdot771anorexia + 0\cdot519mets_{bone} - 0\cdot475dyspnoea - 0\cdot54dysphagia$$

where

| $amts$ | AMTS score (If ≤3 then = 0 , if >3 then = 1) |
| --- | --- |
| $pulse$ | Pulse rate |
| $distant_{mets}$ | Presence of distant metastases (No = 0, Yes = 1) |
| $mets_{liver}$ | Presence of liver metastases (No = 0, Yes = 1) |
| $ecog$ | Eastern Co-operative Oncology Group score |
| $overall\_health$ | Global Health Score |
| $anorexia$ | Anorexia (No = 0, Yes = 1) |
| $mets_{bone}$ | Presence of bone metastases (No = 0, Yes = 1) |
| $dyspnoea$ | Dyspnoea (No = 0, Yes = 1) |
| $dyshpagia$ | Dyshpagia (No = 0, Yes = 1) |

The corresponding probability of survival for the PiPS-A14 model is

$$PiPS_{A_{14}}= \frac{1}{1+exp(-LO_{A_{14}})}$$

**PiPS-A56 model**

The PiPS-A56 log odds are

$${LO}_{A_{56}} = 0\cdot471 + 0\cdot851amts - 0\cdot022pulse -0\cdot407distant_{mets} - 0\cdot596mets_{liver} - 0\cdot219Ecog - 0\cdot421anorexia + 0\cdot549overall\_health + 0\cdot617primary\_breast + 1\cdot477mgo\_cancer - 0\cdot51lost\_weight$$

where

| $amts$ | AMTS score (If ≤3 then = 0, if >3 then = 1) |
| --- | --- |
| $pulse$ | Pulse rate |
| $distant\_mets$ | Presence of distant metastases (No = 0, Yes = 1) |
| $mets liver$ | Presence of liver metastases (No = 0, Yes = 1) |
| $ecog$ | Eastern Co-operative Oncology Group score |
| $Overall\_health$ | Global Health Score |
| $Primary\_breast$ | Primary cancer breast (No = 0, Yes = 1) |
| $Mgocancer$ | Primary cancer Male Genital Organs (Prostate) (No = 0, Yes = 1) |
| $Lost weight$ | Lost weight (No = 0, Yes = 1) |

The corresponding probability of survival for the PiPS-A56 model is:

$$PiPS_{A_{56}}= \frac{1}{1+exp(-LO_{A_{56}})}$$

**PiPS-B14 model**

The PiPS-B14 log odds are:

$${LO}_{B_{14}} = 4\cdot577 + 0\cdot952amts - 0\cdot017pulse - 0\cdot835distant\_mets + 0\cdot767mets_{bone} - 0\cdot678Anorexia - 0\cdot531ecog + 0\cdot393overall\_health -0\cdot061wbc + 0\cdot003platelet - 0\cdot058urea - 0\cdot004alanine -0\cdot006creactive$$

where

| $amts$ | AMTS score (If ≤3 then = 0 if >3 then = 1) |
| --- | --- |
| $pulse$ | Pulse rate |
| $distant_{mets}$ | Presence of distant metastases (No = 0, Yes = 1) |
| $mets_{bone}$ | Presence of bone metastases (No = 0, Yes = 1) |
| $Anorexia$ | Anorexia (No = 0, Yes = 1) |
| $Ecog$ | Eastern Co-operative Oncology Group score |
| $Overall\_health$ | Global Health Score |
| $Wbc$ | WBC (x10^9/L) |
| $Platelet$ | Platelet count (x10^9/L) |
| $urea$ | Urea (mmol/L) |
| $alanine$ | ALT (U/L) |
| $creactive$ | CRP (mg/L) |

The corresponding probability of survival for the PiPS-B14model is:

$$PiPS_{B_{14}}= \frac{1}{1+exp(-LO_{B_{14}})}$$

**PiPS-B56 model**

The PiPS-B56 log odds are:

$${LO}_{B_{56}} = -0\cdot075 -0\cdot013pulse - 0\cdot042wbc + 0\cdot001platelet - 0\cdot031neutrophil + 0\cdot163lymphocyte_{10exp9} - 0\cdot062urea - 0\cdot001alkaline+ 0\cdot040albumin - 0\cdot007creactive + 1\cdot56mgo\_cancer - 0\cdot673fatigue +0\cdot474overall\_health$$

where

| $Pulse$ | Pulse rate |
| --- | --- |
| $Wbc$ | WBC (x10^9/L) |
| $Platelet$ | Platelet count (x10^9/L) |
| $Neutrophil$ | neutrophils (x10^9/L) |
| $lymphocyte_{10exp9}$ | Lymphocytes (x10^9/L) |
| $Urea$ | Urea (mmol/L) |
| $Alkaline$ | Alk Phos (U/L) |
| $Creactive$ | CRP (mg/L) |
| $Mgo\_cancer$ | Primary cancer Male Genital Organs (Prostate) (No = 0, Yes = 1) |
| $fatigue$ | Fatigue (No = 0, Yes = 1) |
| $Overall\_health$ | Global Health Score |

The corresponding probability of survival for the PiPS-B56 model is:

$$PiPS_{B_{56}}= \frac{1}{1+exp(-LO_{B_{56}})}$$
