# Supplementary material for: The Prognosis in Palliative care Study II (PiPS2): A prospective observational validation study of a prognostic tool with an embedded qualitative evaluation
Source: PLoS One. 2021 Apr 28;16(4):e0249297. doi: 10.1371/journal.pone.0249297 (PMC8081241; doi:10.1371/journal.pone.0249297)
Supplement: S2 File — (DOCX) [file pone.0249297.s002.docx]

# S2 File. Topic Guides

## Patients and Carers

**At the start**

We have approached you to ask you to share your experiences of being approached and/or involved with the Prognosis in Palliative Care Study. We would also like to ask your views about the development of a prognosis tool for palliative care patients and what you think about doctors and nurses using prognostic tools with patients?

Where necessary, prompts will be used to guide the discussion.

**Sample Questions**

• I wondered if I could start by asking you a little about your illness/or the illness of your relative/the person you are caring for?

• Can you tell me about some of the reasons why you or your relative or the person you care for decided to or not to take part in the prognostic study?

• Was the decision made following discussion with others around you/them?

• What opinions do you have about the development of a tool/indicator that can estimate the life expectancy of palliative care patients?

• If we could develop a method/tool that is similar or better than doctors’ predictions of life expectancy, would you want it?

• Should health professionals check what prognostic information patients/carers desire before initiating prognostic conversations? (Respecting patients’/carers’ information wishes).

• Do you belief that Health Professionals should always share prognostic information with patients to help patients/family carers make decisions and plans? Why/Why not?

• If it is no more accurate than clinicians’ predictions, is it a useful way of starting the conversation with palliative patients/their carers about life expectancy?

• What do you think is the best and most sensitive way to present prognostic information to patients and or relatives/carers? For example: How do you think the tool/indicator should be introduced to patients/relatives/carers? Prompt: Over time, in writing, via face-to-face conversations.

• Do you think patients/carers would prefer/find it easier to understand if the prognostic tool/indicator said: Probability 1) A patient/the person you care for have X% probability of surviving for X amount of time OR length of survival (precise) 2) The patient/the person you care for are predicted to live for this long? (number of days, weeks, months or years) or Length of survival (vaguer) 3) The outlook for patients like you/the person you care for are: good, bad, average?

• Do you think patients would be more comfortable receiving prognostic information in relation to the probability of reaching a significant future event?

• What are your opinions about the usefulness of such a tool/indicator?

• Do you think there are any disadvantages for patients or carers? If yes, can you tell me about what they are?

• Do you think most palliative care patients and their carers want to know this information?

## Clinicians

**At the start**

We have approached you to take part in this research to share your views and opinions about giving prognostic information to patients/carers as well as using prognostic tools to possibly improve the delivering of survival length prognosis to patients/carers with advanced cancer.

• Can you tell me about your clinical role and how long you have worked in palliative care?

• What are your experiences of making predictions of survival length to patients, relatives or carers?

• Do you discuss with patients/carers how accurate/inaccurate survival predictions can be?

• How easy or difficult do you find it to predict length of survival?

• What information do you share with patients/carers about prognosis and how do you convey it?

• How do you feel delivering such news to relatives or carers? Prompt: Uncomfortable, stressful, etc. Is there anything that makes it easier? More difficult?

• If patients/carers don’t specifically ask about the time they/patients have left do you share or not your estimate of survival time?

• If patients do specifically ask about the time they have left – how do you respond?

• How accurate do you find the estimates that you make?

• Do you/health professionals document in patient’s notes the length of time they have left?

• Are professional guidance/standards available for clinicians on the best way of approaching prognostic discussions?

• Do you check and discuss with patients/carers first if they want prognostic information and what type of information they would prefer, such as the probabilities of cure, survival rates, general expected outcome of the disease?

**Next, I would like to start by showing you some prognostic models/tools:**

• What are you first impressions of the models/tools?

• Clinical usefulness

- - Ease of completion
  - Interpretability of outputs

**Barriers and facilitators to clinical use**

• If the tool is no more accurate than clinicians’ predictions is it something that you would use? Prompt. Could it be a useful way of having that conversation with patients/carers?

• If such a tool was subject to the same clinical errors in judgement would you use it or would you just relay on your own clinical judgement?

• How would you explain to patients the probability/statistics of survival rates?

• If such a tool absolved you of responsibility of delivering inaccurate prognostic information to patients/carers would that make it more acceptable for you to use?

• Would you consider using these types of prognostic tools in day to day clinical practice? Prompt: Why? if yes/no.

• What processes and structures in your work place would help or hinder you using such tools?
