# Supplementary material for: The Prognosis in Palliative care Study II (PiPS2): A prospective observational validation study of a prognostic tool with an embedded qualitative evaluation
Source: PLoS One. 2021 Apr 28;16(4):e0249297. doi: 10.1371/journal.pone.0249297 (PMC8081241; doi:10.1371/journal.pone.0249297)
Supplement: S3 File — (DOCX) [file pone.0249297.s003.docx]

# S3 File. Multiple imputation analysis for PiPS-B model

The PiPS-B model requires blood test results for its calculation. For patients with capacity, a blood sample was obtained. For patients without capacity, blood tests results were only available if the patient had had a blood specimen collected as part of their routine clinical care within 72 hours of study entry. A total of 1610 patients with capacity consented to participate in the study. A further 61 participants, without capacity, had blood results obtained as part of routine clinical care. Consequently, we were potentially able to calculate PiPS-B values for a maximum of 1671 participants. In fact, we only had the complete data required to calculate PiPS-B model values on 1484 patients (1423 participants with capacity and 61 participants without capacity). Consequently, we had complete PiPS-B data on 89% (1484/1671) of those participants who were potentially available to have a PiPS-B value calculated.

In the statistical analysis plan, we had pre-specified that, if the proportion of missing data to calculate the PiPs-B model was greater than 10%, then we would use multiple imputation for the primary analysis of the PiPS-B model. Given the proportion of missing data was, in fact, 11% we have used multiple imputation based on chained equations, to impute missing predictor values.

The imputation model for missing values included the predictors in the PiPS-B risk model; the AMTS (the only predictor of missingness); and centre (given that there was variation in the proportion of missing data across the centres). Additionally, the intra-cluster correlation coefficient (ICC), for the setting was 0.21 (95% Confidence interval 0.05 to 0.57). We used Rubin’s rule to combine the performance measures for the c-statistic, calibration intercept and slope. For the McNemar’s test using the imputed data sets, it was necessary to pool the p-values.

We used ten imputed data sets. Predictive mean matching imputation was used for the continuous and ordinal values and logistic regression was used for the binary outcomes. To represent the centres we used the setting (inpatient, community or hospital).

Discrimination and calibration of PiPS-B14 and PiPS-B56 using imputed data

The discrimination and calibration of the PiPS-B models are shown in Table S2 below. The calibration of the prognostic models was assessed using the calibration intercept and slope based on a logistic regression model fitted to the validation data using the predicted log-odds as the only predictor.

**Table S5: C-statistic for PiPS-B14 and PiPS-B56 models using the imputed dataset**

|  | **C-statistic (95% CI)** | **Calibration intercept (95% CI) *** | **Calibration slope (95% CI) *** |
| --- | --- | --- | --- |
| PiPS-B14 | 0·836 (0.810 to 0.861) | 0.048 (-0.165, 0.262) | 0.792 (0.689, 0.895) |
| PiPS-B56 | 0.808 (0.787 to 0.829) | 0.131 (0.018, 0.245) | 0.900 (0.798, 1.00) |

Accuracy of PiPS-B combined model with imputed data against AMPES

McNemar’s test was used to compare the proportion of overall patient deaths predicted correctly by PiPS-B, in the imputed dataset) with the corresponding proportion predicted correctly by clinicians. There was no statistically significant difference (p=0.875) between the proportion of patient deaths predicted correctly by PiPS-B combined model and the corresponding proportion predicted correctly by clinicians. The 2x2 tables are not presented since the results are based on the calculation of the p-value across 10 imputed datasets.
