# Supplementary material for: The Prognosis in Palliative care Study II (PiPS2): A prospective observational validation study of a prognostic tool with an embedded qualitative evaluation
Source: PLoS One. 2021 Apr 28;16(4):e0249297. doi: 10.1371/journal.pone.0249297 (PMC8081241; doi:10.1371/journal.pone.0249297)
Supplement: S4 File — (DOCX) [file pone.0249297.s004.docx]

S4 File. Qualitative sub-study summary

The primary aim of the nested qualitative research study was to assess the acceptability of the prognostic models to patients, caregivers and clinicians. The secondary objectives were to understand how clinicians discuss prognostic information with patients and relatives/caregivers, from both the patients’ and clinicians’ perspectives.

**Methods**

A purposive sample of patients, caregivers and clinicians who had been asked to participate in the quantitative study in the Manchester and Derby areas (hospice, community and hospital) were invited to participate in the qualitative interview study.

Twenty nine patients and 20 caregivers were recruited; all were relatives of the patients. The majority of patients (25/29; 86%) were recruited from two hospices in the Greater Manchester area. Patients had received a cancer diagnosis and were eligible for enrolment into the PiPS validation study. Five patients had declined to take part in the validation study; their reasons for refusal were explored during the interview. A smaller number of patients (n=4) were recruited from hospital day care, and were receiving palliative care in a rural community Trust. A total of 32 clinicians were recruited. The clinician sample was pragmatic and comprised of health professionals (HPs) who routinely cared for and made prognostic predictions, such as palliative care specialists, oncologists, nurses and GPs. Interview data were entered into NVivo 10 and analysed using the five stages of Framework Analysis: familiarisation, developing a thematic framework, indexing, charting, mapping and interpretation.

**Findings from the patient and caregiver interviews**

The following themes were identified: Desire for detailed prognostic information, Acceptability of PiPS predictor models, and Preferred presentation of sensitive information.

***Theme 1 Desire for detailed patient prognostic information***

The majority of patients and caregivers expressed a desire for detailed information from clinicians about life expectancy. Few reportedly received this information however, suggesting that when information was given, it was not expressed clearly. A number of patients who had asked about their life expectancy commented that doctors tended to be vague, over optimistic and unwilling to deliver news that was considered bad or uncertain. The main reason both patients and caregivers gave for wanting more detailed prognostic information was to make plans and sort out family affairs. This related to both individual and family finances and making funeral arrangements.

***Theme 2 Acceptability of PiPS predictor models***

All participants considered that PiPS models were acceptable for use in clinical practice and a useful aid in helping clinicians to more accurately predict life expectancy. Patients and caregivers also considered that PiPS models could help doctors initiate sensitive conversations about prognostication, helping them to overcome reported reluctance to do so.

***Theme 3 Preferred presentation of sensitive information***

Patients and caregivers agreed that the most appropriate way to receive prognostic information was verbally during face-to-face discussions. Patients and caregivers preferred doctors to convey prognostic information truthfully, honestly and sensitively, with life expectancy presented as days, weeks or months.

**Findings from the Clinician interviews**

Themes from the clinicians’ interviews were: Challenges and difficulties with predicting length of survival, Language used when discussing prognosis, Reasons for over estimating prognosis, Clinicians’ acceptability of PiPS models, Facilitators for use in clinical practice, and Barriers to use in clinical practice.

***Theme 1 Challenges and difficulties with predicting length of survival***

All clinicians commented on the complex nature of estimating length of survival for patients with advanced cancer, and many found it difficult. A number described how challenging it was to predict survival in ‘well’ patients that had not started to deteriorate. They would generally explain to patients and caregivers that accurately predicting length of survival was in many cases an impossible task due to the level of uncertainty involved.

***Theme 2 Language used when discussing prognosis***

Clinicians were asked about the type of language they used when discussing prognostication with patients and caregivers. The majority, irrespective of experience or seniority, tended to avoid giving detailed information with specific timeframes. Both junior and senior clinicians considered that it could cause psychological harm to patients and caregivers, especially once patients had survived beyond their prediction. Insights from this theme suggest that the ‘vague’ language that some patients and caregivers dislike, is used by clinicians because they consider it to be a more honest response when so many factors are at play. There is also some reticence about giving a number in case a patient becomes fixated on it.

***Theme 3 Reasons for over estimating prognosis***

Clinicians were asked about the accuracy of estimating length of survival for patients with advanced cancer, because studies suggest they tend to give inaccurate and over optimistic prognostic information. All agreed that the main reason for overestimating survival was to convey kinder, more optimistic information, perceived as less harmful. They also considered that it was better not to challenge patients’ or caregivers’ perceptions of the patient’s disease trajectory. They described not wanting to dash people’s hope if they were using denial as an effective coping mechanism.

***Theme 4 Clinicians’ acceptability of PiPS models***

When asked about the acceptability of PiPS models, clinicians gave a number of positive responses. The majority considered the models to be useful algorithms that could offer a more scientific approach to estimating patients’ prognoses, than clinical judgement or a hunch.

Anticipating that predictions using the PiPS models could be similar to clinicians’ estimates, we asked whether the models might still be beneficial. Clinicians stated that modelling could both improve their confidence in making accurate predictions of life expectancy, and be a useful communication prompt for conveying prognostic information; it could facilitate starting, and holding, difficult conversations. Some also suggested that PiPS would be helpful for encouraging clinicians to give more realistic predictions, by alleviating responsibility for the prediction from clinicians themselves.

***Theme 5 Facilitators for use in clinical practice***

All clinicians commented that the online PiPS prognosticator tool was user friendly and quick and easy to use. A number of clinicians considered that PiPS could be used as an educational tool, especially for less experienced staff. Participants further commented on how PiPS could help inform decision making, in relation to treatment options and/or planning discharge and care packages. A trainee doctor (F2) explained that PiPS could also support junior doctors with convincing more senior colleagues about a patient’s poor prognosis.

***Theme 6 Barriers to use in clinical practice***

A minority of participants considered it was not appropriate to use PiPS, especially if blood tests were needed to improve the accuracy of the prediction. This related both to the practicalities of organising blood tests for patients with advanced cancer, and the ethical implications of drawing blood from unwell patients. A few GPs considered that introducing PiPS into clinical practice could be time consuming: both completing the tool and finding time to support patients emotionally. Another, potentially more important barrier however was the difficulty with balancing a kinder more optimistic stance, with being realistic with patients considered not ready for receiving devastating news. If PiPS is perceived to potentially do more harm than good, it is unlikely to be used.

**Conclusion**

The nested qualitative sub-study found that patients and their caregivers were generally in favour of having access to more accurate prognostic information and preferred the PiPS method of couching prognostic estimates in terms of whether individuals were expected to survive for “days”, “weeks” or “months+”. Clinicians were generally in favour of using PiPS even if it were no better than an agreed multi-professional survival estimate, because it could have other benefits in terms of communication, training, supporting junior colleagues and providing objective information for the commissioning of care packages. Some clinicians expressed the view that they would be reluctant to use PiPS-B because they preferred to use their own clinical judgement or because they did not believe that taking a blood specimen was appropriate in these circumstances. For the clinician, using a prognostic tool like PiPS would be more akin to giving a patient the results of a test. This would remove some of the direct responsibility for the “bad news” from the clinician and may make it easier for them to broach the subject. Nonetheless, the clinician would still need excellent communication skills to convey this information in a sensitive way and therefore if PiPS were to be used in this way it is clear that clinicians would need to develop good communication skills for this approach to be successful.
