# Supplementary material for: The Prognosis in Palliative care Study II (PiPS2): A prospective observational validation study of a prognostic tool with an embedded qualitative evaluation
Source: PLoS One. 2021 Apr 28;16(4):e0249297. doi: 10.1371/journal.pone.0249297 (PMC8081241; doi:10.1371/journal.pone.0249297)
Supplement: S1 Table — (DOCX) [file pone.0249297.s005.docx]

# S1 Table - Participating units and Principal Investigators

| **Name of site (n=28)** | **Principal Investigator** |
| --- | --- |
| Birmingham St Mary's Hospice | Dr Christina Radcliffe |
| Bronglais General Hospital | Dr Gokulkrishnan Lingesan |
| Coventry & Warwickshire Partnership NHS Trust | Claire Plump |
| University Hospitals of Derby and Burton NHS Foundation Trust | Professor Vaughan Keeley |
| Douglas Macmillan Hospice | Dr Claire Hookey |
| Camden & Islington community palliative care team | Dr Sarah Yardley |
| John Eastwood Hospice | Dr Alpna Chauhan |
| King's College Hospital | Dr Matthew Maddocks |
| St Gemma’s Hospice, Leeds Community Healthcare NHS Trust | Professor Michael Bennett |
| LOROS Hospice | Professor Christina Faull |
| Marie Curie West Midlands Hospice | Dr Claire Ferguson |
| Norfolk Community Health & Care NHS Trust | Dr Katie Soden (Carpenter) |
| Nottingham University Hospital | Dr Andrew Wilcock |
| Phyllis Tuckwell Hospice (Farnham) | Dr Joanna Vriens |
| Pilgrims Hospice | Dr Stephen Cox |
| Princess Alice Hospice | Dr Jennifer Todd |
| Royal Surrey County Hospital NHS Foundation Trust | Dr Andrew Davies |
| St Andrew's Hospice | Dr Jason Boland |
| St Ann's Hospice (Heald Green) | Dr Ashique Ahamed |
| St Ann's Hospice (Little Hulton) | Dr Ashique Ahamed |
| St Catherine's Hospice | Dr Amanda Gregory |
| St George's Healthcare NHS Trust | Dr Ollie Minton |
| St Giles Hospice (Walsall) | Katie Burbridge (Taroni) |
| St Giles Hospice (Whittington) | Katie Burbridge (Taroni) |
| St Richard's Hospice | Dr Nicola Wilderspin / Dr Sarah Onions |
| Sue Ryder Leckhampton Hospice | Dr Paul Perkins |
| University College London Hospital (UCLH)* | Dr Sarah Yardley |
| Worcestershire Royal Hospital | Dr Nicola Heron |

**Notes for S1 Table:**

* UCLH was withdrawn from the study
