# Supplementary material for: The Prognosis in Palliative care Study II (PiPS2): A prospective observational validation study of a prognostic tool with an embedded qualitative evaluation
Source: PLoS One. 2021 Apr 28;16(4):e0249297. doi: 10.1371/journal.pone.0249297 (PMC8081241; doi:10.1371/journal.pone.0249297)
Supplement: S1 Appendix — (DOCX) [file pone.0249297.s006.docx]

S1 Appendix: PiPS2 Investigators’ group – names of authors for referencing in PubMed

| A | Ahamed |
| --- | --- |
| M | Bennett |
| JW | Boland |
| A | Chauhan |
| S | Cox |
| A | Davies |
| C | Faull |
| C | Ferguson |
| A | Gregory |
| N | Heron |
| C | Hookey |
| G | Lingesan |
| M | Maddocks |
| O | Minton |
| S | Onions |
| P | Perkins |
| C | Radcliffe |
| K | Taroni |
| J | Todd |
| J | Vriens |
| A | Wilcock |
| S | Yardley |
